# *Zingiber officinalis* and *Eucalyptus globulus*, Potent Lethal/Repellent Agents against *Rhipicephalus bursa*, Probable Carrier for Zoonosis

**Published:** 2019-06-24

**Authors:** Samin Madreseh-Ghahfarokhi, Amir Dehghani-Samani, Yaser Pirali, Azam Dehghani-Samani

**Affiliations:** 1 Department of Clinical Sciences, Faculty of Veterinary Medicine, Ferdowsi University of Mashhad, Mashhad, Iran; 2 Faculty of Medicine, Birjand University of Medical Sciences, Birjand, Iran; 3 Department of Clinical Sciences, Faculty of Veterinary Medicine, Shahrekord University, Shahrekord, Iran; 4 Department of Pathobiology, Faculty of Veterinary Medicine, Shahrekord University, Shahrekord, Iran; 5 Faculty of Veterinary Medicine, Shahrekord University, Shahrekord, Iran

**Keywords:** Acaricidal activity, Essential oil, Eucalyptus, Ginger, Repellent activity

## Abstract

**Background:**

*Rhipicephalus bursa* is a hard tick with importance in transmission of tick-borne diseases and zoonosis. Natural products are excellent alternative to pesticides. In this study for the first time, lethal and repellent activity of *Zingiber officinalis* and *Eucalyptus globulus* against *Rh. bursa* were studied.

**Methods:**

In July till September of 2017, essential oils were extracted in Shahrekord University, Iran from fresh plant materials and engorged mature ticks were collected from infested sheep herd located in Saman, Iran. Ticks were challenged by different concentrations of essential oils including 300, 500 and 800µl/ml and 1 (pure) singly and/or in combination. Percentages of killed and repellent ticks as efficacy of acaricidal and repellent activity of essential oils against *Rh. bursa* were calculated and analyzed statistically.

**Results:**

Efficacy of eucalyptus essential oils was more in whole concentrations and its highest performance was observed in concentration 1 (pure). Efficacies of ginger and combined essential oils were different for each concentration but their highest efficacies were observed in concentration 1 (pure), too.

**Conclusion:**

This study showed considerable values of acaricidal and repellent activity against *Rh. bursa* for both essential oils singly and in combination, so they can be considered as potent lethal/repellent agents for control of ticks, but more studies need for this purpose, study on lethal/repellent activity of these essential oils and/or other plants against other important arthropods can be considered as subjects for next experiments.

## Introduction

Ticks can transfer some of important diseases that named tick-borne diseases (1). *Rhipicephalus bursa* is a hard tick from the genus Rhipicephalus which is the fourth largest in the Family Ixodidae and has an important role in transferring of different diseases (2). Sheep, goats, cattle, horses are the preferred hosts of *Rh. bursa*, it is a two-host species, usually has a mono-tropic type of behavior, with immature stages feeding on the same host species as the adult tick stages. However, the immaturestages can also feed on rodents and rabbits (3).

*Rhipicephalus bursa* is an important member of common tick fauna in different part of Iran (4–6) and several important microorganisms including: Crimean Congo hemorrhagic fever (CCHF) virus (7), Ehrlichiosis bacteria (8), *Anaplasma phagocytophilum* (9), *Rickettsia aeschlimannii* (10) and other bacteria (11) were isolated repeatedly from *Rh. bursa*.

Use of chemical pesticide agents is increasing every day in order to control ticks populations. About 2.5 million tons of pesticides are used on crops each year and the worldwide damage caused by pesticides reaches $100 billion annually. The reasons for this are: (a) the high toxicity and non-biodegradable properties of pesticides and (b) the residues in soil, water resources and crops that affect public health (12).

Addition to environmental concerns, there are several reports about insecticide-resistant against chemical pesticides like Pyrethroid and Propetamphos in ticks including *Rh. bursa* (13, 14). Thus, searching for replace of chemical pesticides is necessary in order to firstly solve the problem of long term toxicity to mammals and secondly to find environmental friendly pesticides and develop techniques used to reduce pesticide use while maintaining crop yields. Natural products are an excellent alternative to chemical insecticides.

Several plants contain compounds that they use in preventing attack from arthropods and/or insects. These chemicals fall into several categories, including repellents, feeding deterrents, toxins, and growth regulators (15).

Ginger (rhizome of *Zingiber officinale* Roscoe) is one of the most widely used herbal medications in oriental medicine against pain, inflammation, stomach problems, nausea, vomiting, epilepsy, sore throat, cough, common cold, bruises, wounds, liver complaints, rheumatism, muscular pains, atherosclerosis, migraine headaches, high cholesterol, ulcers, and etc. (16). Ginger essential oil can produce from fresh rhizomes and it has many efficient effects like antibacterial, antiviral, antifungal and other properties (17, 18).

Moreover, the genus *Eucalyptus* that knows by over 700 species distributed throughout the world (19), provides variety of components extracted from its essential oil known as insecticide and repellent agent (20). Antimicrobial and antioxidant activity of eucalyptus essential oil were reported (21, 22), also effect of eucalyptus essential oil on respiratory bacteria and viruses, traditional use of eucalyptus in treatment of rhino-sinusitis and anti-diabetic effect of eucalyptus were identified (23–25). There is no study on lethal and repellent activity of *Z. officinalis* and *E. globulus* essential oils against *Rh. bursa*, and this is the first survey in this way. In this study acaricidal and repellent activity of essential oils of ginger and eucalyptus against *Rh. bursa* as a wide-distributed species of ticks in different parts of Iran with an important role in transmission of zoonosis were studied singly and in combination with together.

## Materials and Methods

### Extraction of essential oils

Current study were done from July till September of 2017, fresh rhizomes of *Z. officinalis* and leafs of *E. globulus* were used to essential oil extraction. Fresh rhizomes of *Z. officinalis* were prepared from the shops and fresh leafs of *E. globulus* were collected in summer (July 2017) from grown trees located in Najafabad City, Isfahan Province in center of Iran (32°50′43″N 51°36′00″E). Essential oils extractions were done separately for each sample via conventional hydrodistillation method. Hydrodistillation in a Clevenger-type apparatus consists of immersing the ground plant material directly in a flask filled with water that is then brought to the boil. Vapors carry volatile compounds and the condensate drops on the pentane/ether trap in the inner tube of the apparatus where the volatiles are retained. In current study, 200gr of each sample were crushed and added to 800ml distilled water in a round bottom flask. The flask was heated and the Clevenger apparatus was attached. The mixture was boiled at 100 °C and then the temperature was reduced to 60 °C and kept for 3 h, the recovered mixture was allowed to settle and finally essential oil was withdrawn for each sample separately (26).

### Collection of ticks

Mature engorged male and/or female ticks (disregard of their sex) were collected from a highly infested sheep herd located in the Saman City, Chaharmahal and Bakhtiari Province, southwest of Iran (32°27′06″N 50°54′ 38″E). Infestation was so high in the herd and ticks were mostly presented around or inside of outer ear, perineal area and around the vent and under the groin area, ticks dis-attaching was done via sterile forceps, without any palpation and with the low pressure of forceps and their rotation. Guard glass, disposable dress, and multi-layer disposable gloves were used during the work and whole the health concerns were considered. In order to species-specific collecting, morphological aspects of *Rh. bursa* species like color were considered during the collecting procedure. Collected ticks were immediately transferred to specific glass boxes, kept in cool place away from any chemical agents and sunlight, transferred to laboratory for experiment. Note that dis-attaching of ticks was a difficult procedure and some ticks were injured during the collecting or transferring, that they were removed for experiment. Totally, about 700 ticks were collected. Confirmed species identification was done under the laboratory optic loop via identification keys for tick’s species (3), also health of ticks was controlled again under the loop and probable injured ticks were removed. Differentially, 93 % of collected ticks were identified as *Rh. bursa* and about 7% were identified as other species of hard ticks. Finally, 640 engorged health ticks were prepared for experiment, in order to deletion of biases in experiment, collected ticks were immediately examined after collection and till the experiment, ticks were incubated in 25 °C and 80% humidity, in common glass box with air circulation.

### Treatments preparation

Concentrations of 300, 500 and 800µl/ml and 1 (pure) were prepared from essential oils of *E. globulus* and *Z. officinalis* separately via combination by different amount of normal saline, also mixed concentrations of 150µl/ml Eucalyptus plus 150µl/ml ginger, 250µl/ml Eucalyptus plus 250µl/ml ginger, 400µl/ml Eucalyptus plus 400µl/ml ginger and 500 µl/ml Eucalyptus plus 500µl/ml ginger were prepared with the same method. In order to sure of tests validity, a positive control group with concentration of 100mg/ml was prepared via dilution of 25ml of Ripcord ® (Cypermethrin 40%, Spiagri Company, Tehran, Iran) in 75ml normal saline, also a negative control group was prepared from pure normal saline without any additive components.

### Evaluation of acaricidal activity of essential oils

Three replication tests were done for every treatment (including positive and negative control groups) and for each replication 13 health ticks were transferred to plastic nets and dipped in different concentrations of essential oils, and/or positive and negative control solution, with same temperature (25 °C) for 30sec, then they were transferred to specific glass dishes with same condition including humidity, light and air circulation for all of treatments and then were incubated in 25 °C and 80% humidity for two hours. Note that all the methods were same for every groups and replications. After the two hours, percentage of died ticks as acaricidal efficacy of each treatment were counted and mean of efficacy for each treatment were calculated and analyzed statistically. For sure whole of died ticks were carefully observed under the laboratory optic loop.

### Evaluation of the repellent activity of essential oils

Repellent activity of each essential oils against *Rh. bursa* in concentration of 1 (pure) singly and 500µl/ml eucalyptus plus 500µl/ml ginger in combination were studied by Y-tube olfactometer bioassay. Y-tube olfactometer consists of a glass Y-tube with the main arm (the stem) and 2 arms containing one repellent and control in another one, where a low rate air movement is created by sucking the air in the two arms of the Y-tube with a pump connected to the stem. The essential oil sample and control are applied on a paper attached to the arms of the tube. Arthropods are introduced into the tube by a hole located at the center (the joint point of the three tubes). After introduction, the hole is closed with a rubber stopper and the pump is operated. After specific time of exposition, the number of arthropods on each of the 2 tubes (treated and control) are scored to assess the percentage of repellency (27, 28). In this study, triplicate tests were done and in each replication 13 ticks were put into every arm and 10 ml of each mentioned treatments was added into treated tube and nothing into control tube. Hard ticks are slow, so more time considered and five hours after pumping, the number of repellent ticks was reported as repellent percentage of treatment and control groups. Note that after each application of Y-tube olfactometer, it was cleaned, washed and dried for next examination.

### Statistical analysis

The analyzed data were expressed as the Mean±standard error of the mean (SEM) using Sigma stat (ver. 3.1) software. Groups were compared using one-way ANOVA for repeated measurements. A value of (P≤ 0.05) was considered significant.

## Results

### Acaricidal activity of essential oils

Results of current study showed different amounts of efficacy (lethal effect) for different concentration of essential oils, singly and in combination. Highest acaricidal activities against *Rh. bursa* were observed for concentration 1 (pure) of eucalyptus and gingers essential oils singly. The most potent treatment (about 54%) was eucalyptus essential oil in concentration 1 (pure) and the lowest activity (about 8.5%) was observed for combined essential oils in concentration 150+150 µl/ml of essential oils. Significant differences (P≤ 0.05) between different concentrations of eucalyptus essential oil were also observed and the highest increase in its acaricidal activity was observed between concentration 800µl/ml and 1 (pure) of this essential oil. Ginger essential oil also had different values o acaricidal activity in different concentrations, its highest activity was observed in concentration 1 (pure) too, but its activity had equal increase between different concentrations and the highest increase in its acaricidal activity of combined essential oils was observed between concentrations mixed 150+150µl/ml and mixed 250+250µl/ml of essential oils. Table 1 shows different values of acaricidal activity in different concentrations of essential oils singly and in combination.

**Fig. 1.**
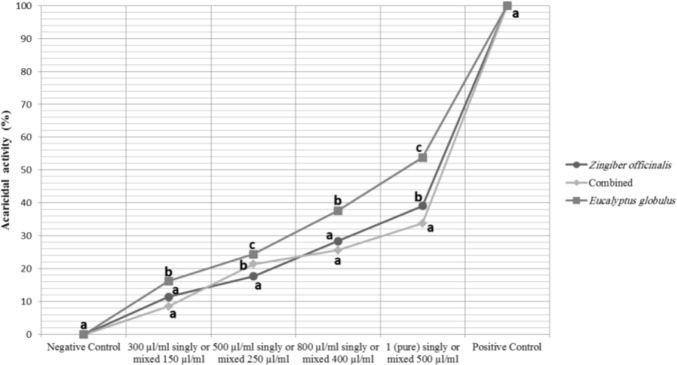
Comparison between acaricidal activities of different treatments in each concentration. Presence of different lowercase letters (a–f) in each concentration shows the significant differences (P≤ 0.05) between different essential oils (lines).

Comparison between efficacies of different essential oil treatments in each concentration shows that eucalyptus essential oils were the most potent agents than others in whole concentrations. Efficacies of ginger essential oils in different concentration were between the efficacies of eucalyptus and combined essential oils except for concentration 500µl/ml that efficacy of combined essential oils was intermediate. Performances of different treatments in each concentration are compared in Fig. 1.

**Fig. 2.**
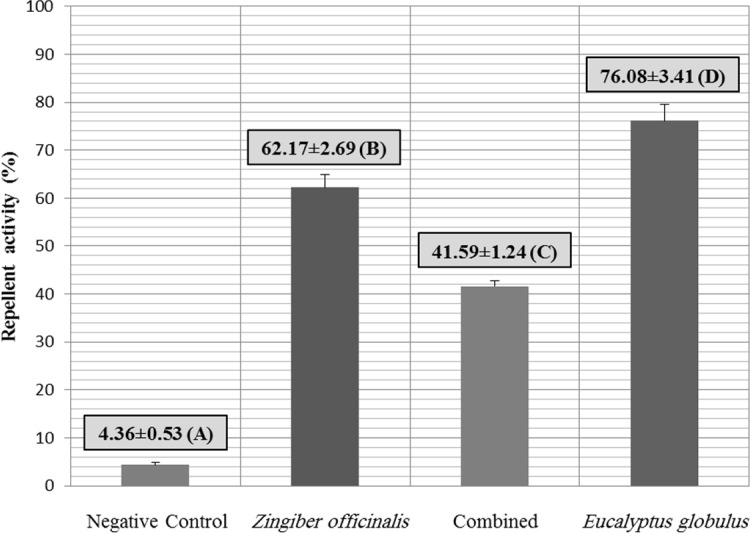
Repellent activities of different essential oils versus control group. Presence of different uppercase letters (A–D) shows the significant differences (P≤ 0.05) between groups.

Different values of repellent activity were observed for different essential oils group versus control group. Eucalyptus essential oil had the highest repellent activity significantly (P≤ 0.05) against *Rh. bursa* and other groups had different efficacies. Comparison between repellent activities of examined groups is shown in Fig. 2.

**Table 1.** Acaricidal activity (Mean±SEM)% of different concentrations for each treatment (essential oils) versus negative and positive control groups

**Groups**	***Z. officinalis***	**Combined**	***E. globulus***
**Negative Control**	0^*^^a^	0^a^	0^a^
**300µl/ml singly or mixed 150µl/ml**	11.47±0.29^b^	8.53±0.86^b^	15.21±1.09^b^
**500µl/ml singly or mixed 250µl/ml**	16.73±1.54^c^	21.38±2.92^c^	24.38±0.42^c^
**800µl/ml singly or mixed 400µl/ml**	28.36±2.09^d^	25.62±1.37^c^	37.65±2.64^d^
**1 (pure) singly or mixed 500 µl/ml**	39.08±1.29^e^	33.75±2.83^d^	53.83±3.14^e^
**Positive Control**	100^f^	100^e^	100^f^

* Presence of different superscript lowercase letters (^a–f^) shows the significant differences (P≤0.05) between different concentrations (rows) of each essential oil treatment (column).

## Discussion

Role of *Rh. bursa* in transmission of many important zoonosis microorganisms (7–11), failure of chemical pesticides and presence of pesticides-resistant ticks (13, 14) and lack of studies on acaricidal and repellent natural products against *Rh. bursa* were the reasons for design of current study. Natural products, such as essential oils produced by the secondary metabolism of herbs and are used in human consumption as functional food, food additives, medicines, nutritional supplements and the manufacture of cosmetics due to their properties (29) are good candidates as replace for chemical products.

There is no similar study about acaricidal and/or repellent activity of essential oil of natural plants like *Z. officinalis* and *E. globulus* against *Rh. bursa*. In this study for the first time acaricidal and repellent activity of essential oils of *Z. officinalis* and *E. globulus* against *Rh. bursa* as a wide-distributed species of ticks in different parts of Iran with an important role in transmission of zoonosis were studied singly and in combination with together.

Acaricidal and repellent activities of eucalyptus essential oil were studied against the poultry red mite, *Dermanyssus gallinae* (Acari: Mesostigmata) and its efficacies were reported about 90% and 94% for acaricidal and repellent activity respectively (20), in current study acaricidal and repellent activities of eucalyptus essential oil against *Rh. bursa* (Acari, Ixodidae) were determined about 54% and 76% respectively, that maybe its lowest performances occurred due to different resistance between these ticks species.

Insecticidal and repellent activity of essential oil of *Z. officinalis* and *E. globulus* against *Culex theileri* Theobald were reported in another study, the highest value of insecticidal activity of eucalyptus essential oil was 66% and its highest repellent activity against *Culex theileri* Theobald was 74% and for ginger essential oil insecticidal and repellent activities was 39% and 62%, respectively (30). Results of current study agreed with them. In current study eucalyptus, essential oil had the highest acaricidal (54%) and repellent (76%) activates against *Rh. bursa*. Ginger essential oil had intermediate effects between treatments and its acaricidal and repellent activities was 39% and 62% respectively and values of 34% and 42% were observed as the lowest results for acaricidal and repellent activities of combined essential oils respectively.

Acaricidal effect of *Pelargonium roseum* and eucalyptus essential oils against adult stage of *Rhipicephalus annulatus* were also studied and dose-dependent effects were reported, highest acaricidal effects were observed for ticks treated with 5% dilutions of *Pelargonium roseum* (79.2%) and eucalyptus (16.7%) after one day incubation (31), that higher value of acaricidal activity of eucalyptus essential oil in current study versus their results maybe was observed due to higher concentration of this essential oil in current study.

Pesticide and/or repellent activity of eucalyptus essential oil against *Acanthoscelides obtectus* (Say) (32), *Culex quinquefasciatus* (33), housefly, *Musca domestica* (34), *Pediculus humanus capitis* (Anoplura:  Pediculidae) (35) and *Lutzomyia longipalpis* (36) were reported. Result of current study agreed with them and shows acaricidal/repellent activity for eucalyptus essential oil due to presence of components such as 1, 8-cineole, citronellal, citronellol, citronellyl acetate, p-cymene, eucamalol, limonene, linalool, a-pinene, g-terpinene, a-terpineol, alloocimene, andaromadendrene in eucalyptus essential oil (37).

There is a little data about insecticidal and/or repellent activity of ginger essential oil. Insecticidal, repellent and oviposition-deterrent activity of ginger essential oil against *Anopheles stephensi* and *Aedes aegypti* (38) and larvae of *Spodoptera littoralis* (39) were reported. Result of current study was agrees with them and showed acaricidal/repellent activity of essential oil of ginger due to presence of presence of bioactive compounds such as gingerols, shogaols, diarylheptanoids, phenylbutenoids, flavanoids, diterpenoids and sesquiterpenoids in it (40).

Moreover, briefly, insecticidal and/or acaricidal effect of *Micromeria fruticosa*, *Nepeta racemosa* and *Origanum vulgare* (Lamiaceae) essential oils against *Tetranychus urticae* and *Bemisia tabaci* (41) were reported in another study, also essential oils of *Cuminum cyminum*, *Pimenta dioica* and *Ocimum basilicum* had different values of acaricidal effects against the cattle tick *Rhipicephalus* (*Boophilus*) *microplus* (42). In another study, acaricidal activity of essential oils of *Lippia graveolens*, *Rosmarinus officinalis*, and *Allium sativum* against *Rhipicephalus microplus* were identified (43). Acaricidal properties of *Artemisia absinthium* and *Tanacetum vulgare* essential oils against *Tetranychus urticae* were also studied (44).

## Conclusion

Briefly, eucalyptus essential oil had the highest acaricidal (54%) and repellent (76%) activates against *Rh. bursa*. Ginger essential oil had intermediate effects between treatments and its acaricidal and repellent activities were 39% and 62% respectively and values of 34% and 42% were observed as the lowest results for acaricidal and repellent activities of combined essential oils respectively. These agents can be considered as potent lethal/repellent agents for control of ticks, but more studies need for this purpose, study on lethal/repellent activity of these essential oils and/or other plants essential oils against other/this important arthropods can be considered as subjects for next experiments.

## References

[1] Bratton RL Corey GR (2005) Tick-borne disease. Am Fam Physician. 71(12): 2323–2330. 15999870

[2] Noori NV Rahbari S Bokaei S (2012) The seasonal activity of *Rhipicephalus bursa* in Cattle in Amol (Northern Iran). World Appl Sci J. 18: 590–593.

[3] Walker AR (2003) Ticks of domestic animals in Africa: a guide to identification of species. Bioscience Reports, Edinburgh.

[4] Razmi GR Glinsharifodini M Sarvi S (2007) Prevalence of ixodid ticks on cattle in Mazandaran Province, Iran. Korean J Parasitol. 45(4): 307–310. 18165714 10.3347/kjp.2007.45.4.307 PMC2532622

[5] Yakhchali M Hosseine A (2006) Prevalence and ectoparasites fauna of sheep and goats flocks in Urmia suburb, Iran. Vet arhiv. 76(5): 431–442.

[6] Rahbari S Nabian S Shayan P (2007) Primary report on distribution of tick fauna in Iran. Parasitol Res. 101 Suppl 2: S175–7. 17823823 10.1007/s00436-007-0692-7

[7] Telmadarraiy Z Ghiasi SM Moradi M Vatandoost H Eshraghian MR Faghihi F Zarei Z Haeri A Chinikar S (2010) A survey of Crimean-Congo haemorrhagic fever in livestock and ticks in Ardabil Province, Iran during 2004–2005. Scand J Infect Dis. 42(2): 137–141. 19958240 10.3109/00365540903362501

[8] Masala G Chisu V Foxi C Socolovschi C Raoult D Parola P (2012) First detection of *Ehrlichia canis* in *Rhipicephalus bursa* ticks in Sardinia, Italy. Ticks Tick Borne Dis. 3(5–6): 396–397. 23140896 10.1016/j.ttbdis.2012.10.006

[9] Dahmani M Davoust B Rousseau F Raoult D Fenollar F Mediannikov O (2017) Natural Anaplasmataceae infection in *Rhipicephalus bursa* ticks collected from sheep in the French Basque Country. Ticks Tick Borne Dis. 8(1): 18–24. 27666778 10.1016/j.ttbdis.2016.09.009

[10] Fernandez-Soto P Encinas-Grandes A Perez-Sanchez R (2003) *Rickettsia aeschlimannii* in Spain: molecular evidence in *Hyalomma marginatum* and five other tick species that feed on humans. Emerg Infect Diseases. 9(7): 889–890. 12899141 10.3201/eid0907.030077 PMC3023448

[11] Ioannou I Sandalakis V Kassinis N Chochlakis D Papadopoulos B Loukaides F Tselentis Y Psaroulaki A (2011) Tick-borne bacteria in mouflons and their ectoparasites in Cyprus. J Wildl Dis. 47 (2): 300–306. 21441182 10.7589/0090-3558-47.2.300

[12] Isman MB Machial CM (2006) Pesticides based on plant essential oils: from traditional practice to commercialization. Advances Phytomed. 3: 29–44.

[13] Enayati AA Asgarian F Amouei A Sharif M Mortazavi H Boujhmehrani H Hemingway J (2010) Pyrethroid insecticide resistance in *Rhipicephalus bursa* (Acari, Ixodidae). Pest Biochem Physiol. 97 (3): 243–248.

[14] Enayati AA Asgarian F Sharif M Boujhmehrani H Amouei A Vahedi N Boudaghi B Piazak N Hemingway J (2009) Propetamphos resistance in *Rhipicephalus bursa* (Acari, Ixodidae). Vet Parasitol. 162(1–2): 135–141. 19286323 10.1016/j.vetpar.2009.02.005

[15] Maia MF Moore SJ (2011) Plant-based insect repellents: a review of their efficacy, development and testing. Malar J. 10 Suppl 1: S11. 21411012 10.1186/1475-2875-10-S1-S11 PMC3059459

[16] Shukla Y Singh M (2007) Cancer preventive properties of ginger: a brief review. Food Chem Toxicol. 45(5): 683–690. 17175086 10.1016/j.fct.2006.11.002

[17] Singh G Maurya S Catalan C De Lampasona MP (2005) Studies on essential oils, Part 42: chemical, antifungal, antioxidant and sprout suppressant studies on ginger essential oil and its oleoresin. Flavour Fragr J. 20(1): 1–6.

[18] Koch C Reichling J Schneele J Schnitzler P (2008) Inhibitory effect of essential oils against herpes simplex virus type 2. Phytomedicine. 15(1–2): 71–8. 17976968 10.1016/j.phymed.2007.09.003

[19] Brooker MIH Kleinig DA (1994) Field Guide to Eucalyptus. (Third ed). Bloomings Books, Melborne.

[20] Dehghani-Samani A Madreseh-Ghahfarokhi S Dehghani-Samani A Pirali-Kheirabadi K (2015) Acaricidal and repellent activities of essential oil of *Eucalyptus globulus* against *Dermanyssus gallinae* (Acari: Mesostigmata). J Herb Med Pharmacol. 4(3): 81–84.

[21] Vazquez G Fontenla E Santos J Freire MS Gonzalez-Alvarez J Antorrena G (2008) Antioxidant activity and phenolic content of chestnut (*Castanea sativa*) shell and eucalyptus (*Eucalyptus globulus*) bark extracts. Ind Crops Prod. 28 (3): 279–285.

[22] Gilles M Zhao J An M Agboola S (2010) Chemical composition and antimicrobial properties of essential oils of three Australian Eucalyptus species. Food Chemist. 119(2): 731–737.

[23] Guo R Canter PH Ernst E (2006) Herbal medicines for the treatment of rhinosinusitis: a systematic review. Otolaryngol Head Neck Surg. 135(4): 496–506. 17011407 10.1016/j.otohns.2006.06.1254

[24] Cermelli C Fabio A Fabio G Quaglio P (2008) Effect of eucalyptus essential oil on respiratory bacteria and viruses. Curr Microbiol. 56(1): 89–92. 17972131 10.1007/s00284-007-9045-0

[25] Mahmoudzadeh-Sagheb H Heidari Z Bokaeian M Moudi B (2010) Antidiabetic effects of *Eucalyptus globulus* on pancreatic islets: a stereological study. Folia Morphol (Warsz). 69(2): 112–8. 20512762

[26] Blazevic I Mastelic J (2009) Glucosinolate degradation products and other bound and free volatiles in the leaves and roots of radish (*Raphanus sativus* L.). Food Chemist. 113: 96–102.

[27] Geier M Boeckh J (1999) A new Y-tube olfactometer for mosquitoes to measure the attractiveness of host odours. Entomol Exp Appl. 92(1): 9–19.

[28] Koul O Singh G Singh R Singh J (2007) Mortality and reproductive performance of Tribolium castaneum exposed to anethole vapours at high temperature. Biopestic Int. 3: 126–137.

[29] Zengin H Baysal AH (2014) Antibacterial and antioxidant activity of essential oil terpenes against pathogenic and spoilage-forming bacteria and cell structure-activity relationships evaluated by SEM microscopy. Molecules. 19(11): 17773–17798. 25372394 10.3390/molecules191117773 PMC6272013

[30] Madreseh-Ghahfarokhi S Pirali Y Dehghani-Samani A Dehghani-Samani A (2018) The insecticidal and repellent activity of ginger (*Zingiber officinale*) and eucalyptus (*Eucalyptus globulus*) essential oils against *Culex theileri* Theobald, 1903 (Diptera: Culicidae). Ann Parasitol. 64(4): 351–360. 30738419

[31] Pirali-Kheirabadi K Razzaghi-Abyaneh M Halajian A (2009) Acaricidal effect of *Pelargonium roseum* and *Eucalyptus globulus* essential oils against adult stage of *Rhipicephalus (Boophilus) annulatus* in vitro. Vet Parasitol. 162(3–4): 346–349. 19356854 10.1016/j.vetpar.2009.03.015

[32] Papachristos DP Karamanoli KI Stamopoulos DC Menkissoglu‐Spiroudi U (2004) The relationship between the chemical composition of three essential oils and their insecticidal activity against *Acanthoscelides obtectus* (Say). Pest Manag Sci. 60(5): 514–520. 15154521 10.1002/ps.798

[33] Mandal S (2011) Repellent activity of Eucalyptus and *Azadirachta indica* seed oil against the filarial mosquito *Culex quinquefasciatus* Say (Diptera: Culicidae) in India. Asian Pac J Trop Biomed. 1(1): S109–112.

[34] Kumar P Mishra S Malik A Satya S (2012) Compositional analysis and insecticidal activity of *Eucalyptus globulus* (family: Myrtaceae) essential oil against housefly (Musca domestica). Acta Trop. 122(2): 212–218. 22326717 10.1016/j.actatropica.2012.01.015

[35] Yang YC Choi HY Choi WS Clark JM Ahn YJ (2004) Ovicidal and adulticidal activity of *Eucalyptus globulus* leaf oil terpenoids against *Pediculus humanus capitis* (Anoplura: Pediculidae). J Agric Food Chem. 52(9): 2507–2511. 15113148 10.1021/jf0354803

[36] Maciel MV Morais SM Bevilaqua CM Silva RA Barros RS Sousa RN Sousa LC Brito ES Souza-Neto MA (2010) Chemical composition of Eucalyptus spp. essential oils and their insecticidal effects on *Lutzomyia longipalpis*. Vet Parasitol. 167(1): 1–7. 19896276 10.1016/j.vetpar.2009.09.053

[37] Liu X Chen Q Wang Z Xie L Xu Z (2008) Allelopathic effects of essential oil from *Eucalyptus grandis*/*E. urophylla* on pathogenic fungi and pest insects. Frontiers Forestry China. 3(2): 232–236.

[38] Prajapati V Tripathi AK Aggarwal KK Khanuja SP (2005) Insecticidal, repellent and oviposition-deterrent activity of selected essential oils against *Anopheles stephensi*, *Aedes aegypti* and *Culex quinquefasciatus*. Bioresour Technol. 96 (16): 1749–1757. 16051081 10.1016/j.biortech.2005.01.007

[39] Pavela R (2005) Insecticidal activity of some essential oils against larvae of Spodoptera littoralis. Fitoterapia. 76(7–8): 691–696. 16236461 10.1016/j.fitote.2005.06.001

[40] Sivasothy Y Chong WK Hamid A Eldeen IM Sulaiman SF Awang K (2011) Essential oils of Zingiber officinale var. rubrum Theilade and their antibacterial activities. Food Chemist. 124(2): 514–517.

[41] Çalmaşur Ö Aslan İ Şahin F (2006) Insecticidal and acaricidal effect of three Lamiaceae plant essential oils against *Tetranychus urticae* Koch and *Bemisia tabaci Genn*. Ind Crops Prod. 23(2): 140–146.

[42] Martinez-Velazquez M Castillo-Herrera GA Rosario-Cruz R Flores-Fernandez JM Lopez-Ramirez J Hernandez-Gutierrez R del Carmen Lugo-Cervantes E (2011) Acaricidal effect and chemical composition of essential oils extracted from *Cuminum cyminum*, *Pimenta dioica* and *Ocimum basilicum* against the cattle tick *Rhipicephalus* (*Boophilus*) *microplus* (Acari: Ixodidae). Parasitol Res. 108(2): 481–487. 20865426 10.1007/s00436-010-2069-6

[43] Martinez-Velazquez M Rosario-Cruz R Castillo-Herrera G Flores-Fernandez JM Alvarez AH Lugo-Cervantes E. Acaricidal effect of essential oils from *Lippia graveolens* (Lamiales: Verbenaceae), *Rosmarinus officinalis* (Lamiales: Lamiaceae), and *Allium sativum* (Liliales: Liliaceae) against *Rhipicephalus* (*Boophilus*) *microplus* (Acari: Ixodidae). J Med Entomol. 48(4): 822–827. 21845941 10.1603/me10140

[44] Chiasson H Bélanger A Bostanian N Vincent C Poliquin A (2001) Acaricidal properties of *Artemisia absinthium* and *Tanacetum vulgare* (Asteraceae) essential oils obtained by three methods of extraction. J Econ Entomol. 94(1): 167–171. 11233109 10.1603/0022-0493-94.1.167

